# Macrophage Polarization, Metabolic Reprogramming, and Inflammatory Effects in Ischemic Heart Disease

**DOI:** 10.3389/fimmu.2022.934040

**Published:** 2022-07-18

**Authors:** Xiaoqian Sun, Yanqin Li, Qiong Deng, Yueyao Hu, Jianteng Dong, Wei Wang, Yong Wang, Chun Li

**Affiliations:** ^1^ College of Chinese Medicine, Beijing University of Chinese Medicine, Beijing, China; ^2^ Beijing Key Laboratory of Traditional Chinese Medicine (TCM) Syndrome and Formula, Beijing University of Chinese Medicine, Beijing, China; ^3^ Guangzhou University of Chinese Medicine, Guangzhou, China; ^4^ School of Life Sciences, Beijing University of Chinese Medicine, Beijing, China; ^5^ Modern Research Center for Traditional Chinese Medicine (TCM), Beijing University of Chinese Medicine, Beijing, China

**Keywords:** macrophage, polarization, metabolic reprogramming, inflammation, ischemic heart disease

## Abstract

Macrophages are highly plastic cells, and the polarization-activating actions that represent their functional focus are closely related to metabolic reprogramming. The metabolic reprogramming of macrophages manifests itself as a bias toward energy utilization, transforming their inflammatory phenotype by changing how they use energy. Metabolic reprogramming effects crosstalk with the biological processes of inflammatory action and are key to the inflammatory function of macrophages. In ischemic heart disease, phenotypic polarization and metabolic shifts in circulating recruitment and tissue-resident macrophages can influence the balance of inflammatory effects in the heart and determine disease regression and prognosis. In this review, we present the intrinsic link between macrophage polarization and metabolic reprogramming, discussing the factors that regulate macrophages in the inflammatory effects of ischemic heart disease. Our aim is to estabilsh reliable regulatory pathways that will allow us to better target the macrophage metabolic reprogramming process and improve the symptoms of ischemic heart disease.

## Introduction

The concept of metabolic reprogramming was first introduced in the last century, and this metabolic pathway was first discovered by Otto Warburg in his studies of tumor cells. Cells absorb glucose under oxygen, and ATP is quickly generated in glycolysis, resulting in lactic acid accumulation, accompanied by a weakened process of oxidative phosphorylation (OXPHOS) reaction (1). This process is also known as the “Warburg effect”. In subsequent studies (2–4), macrophages were also observed to produce similar Warburg effects, and in the 1970s, Hard et al. first identified differences in the metabolic function of M1 macrophages; since then, this area of research has been explored in depth. In this review, we will systematically discuss the metabolic changes associated with macrophages, the related influencing factors, and the importance of macrophage metabolism in the development of ischemic heart disease.

Macrophages are immune-related cells that exist as the “forerunners” of pathogen removal in the body. They play an essential role in immune response, coordination of tissue internal environmental balance, and repair of local tissue loss (5, 6). In addition, the process of macrophage metabolic reprogramming is inextricably linked to the adjustment of its functional activities. i.e., the metabolites and enzymatic reactions generated the reprogramming; this will feed back to the changes in the internal environment that macrophages are located in, ultimately bringing the functional activities of macrophages into balance.

## Metabolism of Unactivated Macrophages

Like most cells, the metabolic pathway of unactivated macrophages is primarily based on glucose and fatty acid uptake. Aerobic oxidation of glucose and β-oxidation of fatty acids, with a well-developed intracellular tricarboxylic acid (TCA) cycle and mitochondrial OXPHOS, continuously produces ATP for energy supply. Meanwhile, with altered homeostasis *in vivo* and infiltration of associated pathological products as well as exogenous substances, macrophages can activate metabolic patterns associated with them to adapt to such changes. This shift in environmentally adaptive metabolic patterns is known as metabolic reprogramming of macrophages.

## Differences in Macrophage Phenotypes Associated With Metabolic Reprogramming

Macrophages are highly plastic cells derived from bone marrow monocytes. Depending on their transformation into macrophages with different functional focuses, which is derived from their high degree of internal environmental adaptability. Different organs are in relatively independent internal environmental balance, which is responsible for the varying functions of macrophages in various sites. Macrophages in the resting state are called inactive “M0” type and are in unpolarized state. As of the present study, macrophages are broadly divided into M1 and M2 forms based on structural and functional differences, whereas M2 type is divided into four subtypes—M2a, M2b, M2c, and M2d, due to differences in activation conditions (7–9). Macrophage plasticity depends on signals from the cell’s external environment, and different species of macrophage subtypes will reflect metabolic reprogramming with their own focuses (10).

In terms of activation, macrophages named M1 in the classical activation mode are generally induced by microbial or aseptic pro-inflammatory stimuli. It usually includes exogenous lipopolysaccharide (LPS) or cytokine interferon-γ (IFN-γ) secreted by TH-1 lymphocytes, viruses, and exogenous DNA stimulation (11, 12), or the macrophage responses are caused by various types of endogenous organism of damage-related molecular patterns (DAMPs), including various types of tissue injury debris and other stress effector proteins (13). At this time, macrophages will tend toward inflammatory activation in several ways, including energy metabolic utilization, metabolite production, and protein secretion. However, the resulting secretion of pro-inflammatory cytokines alters extracellular homeostasis and intracellular transcription, and this reciprocal network effect feeds back into the persistent M1 polarization until energy metabolic process reaches a dynamic balance with the regulation of cellular functions (14), such as by the regulation of macrophage inflammatory regressive mediators, or is transformed into resolution (15). Similarly, M2 macrophages, i.e., substitution-activated type, perform functions that promote phagocytosis, secrete anti-inflammatory mediators, and accelerate tissue injury repair. Similar to the M1 macrophage activation pathway. The M2 polarization of macrophages can be induced by IL-4 or IL-13 secreted by innate and adaptive immune cells (10, 16). Recent studies have further divided M2 macrophages into more detailed subtypes. Stimulants include glucocorticoids, TLR ligands/IL-1Ra, prostaglandin E2 (PGE2), and immune complexes. Although there are many different types, the overall cellular function is to promote the regression of inflammation.

The polarization activation process of macrophages generates reprogramming action of energy utilization, and macrophages can exhibit diverse polarization functional properties by altering their energy metabolic characteristics.

## Differences in Metabolic Reprogramming Macrophages

Overall, macrophage metabolic reprogramming is a reflection of a shift in their functional activity, and the balance of their phenotypic transition can explain this process. This phenomenon is marked by alterations in multiple metabolic pathways: 1) glycolysis, 2) OXPHOS, 3) TCA cycle, and 4) fatty acid oxidation (FAO) (17). Such metabolic variability is particularly evident in macrophages classified by the M1/M2 approach.

Differences in the metabolism of M1 and M2 macrophages are the consensus reached so far on their metabolism. To discuss the adaptation of macrophage metabolism, we must first start with the adaptation of their functions *in vivo*. The metabolic reprogramming effect accompanying macrophage polarization is essentially a functional adjustment action exhibited after adaptation to the internal and external environment in which they live. Macrophages are immune cells, and metabolic reprogramming is an adaptation of the immune regulation of macrophages focusing on the most direct impact on the intake and utilization of energy substrates.

As we mentioned above, in the resting state, macrophages produce ATP for energy supply mainly through the TCA and OXPHOS (18, 19). In contrast, at least *in vitro* (LPS or LPS + IFN-γ stimulation), different metabolic changes were observed in macrophages. The rate of ATP production is increased by the preferential proceeding of glycolysis (18, 20, 21). In this case, macrophages are induced into the M1 type, when glucose, which has been taken up at an increased rate, enters the cell and undergoes the glycolytic pathway to generate pyruvate. As the production of isocitrate dehydrogenase (IDH) and succinate dehydrogenase (SDH) is inhibited (22), the TCA cycle is interrupted, citric and succinic acid start to accumulate, and so pyruvate is directly converted to lactate instead of acetyl coenzyme A to participate in the TCA cycle. Moreover, the reduction of nicotinamide adenine dinucleotide caused by the inhibition of TCA eventually weakens the OXPHOS process. Meanwhile, during pyruvate production from M1 macrophage glycolysis, the pentose phosphate pathway is promoted to produce equivalents of NADPH, which will participate in fatty acid synthesis together with the accumulated citric acid (23). The latter is used to produce inflammatory mediators such as prostaglandins and leukotrienes and for membrane remodeling processes in cells, which involve intracellular inflammatory signaling. This section will be discussed in detail later in this review.

The energy metabolism of macrophages after M1 polarization has such a key point: With significant inhibition of mitochondrial respiration, ATP synthesis is drastically reduced, and macrophages are in an inflammatory state of activation. Although its production of ATP synthesis is lower than that of TCA, it still requires a basic supply of ATP. One theory is that, unlike tumor cells, aerobic glycolysis in macrophages is reversible. To maintain ATP levels for the cell survival response, aerobic glycolysis “makes up” for the decrease in ATP production (2, 20). It has also been suggested that the ATP produced by glycolysis is a key factor used to maintain the membrane potential and integrity of mitochondria. Because mitochondrial integrity is a prerequisite for the accumulation of succinate and isocitrate when the TCA process is inhibited, rather than a complete disruption of mitochondrial function (2, 24, 25). At the same time, aerobic glycolysis generates ATP and lactate—metabolites that accumulate when the TCA cycle is weakened, which will meet its inflammatory metabolic requirements.

In contrast, M2 macrophages do not undergo aerobic glycolytic processes, and recent studies have indicated that it is controversial whether glycolytic processes are inhibited in M2 macrophages (26, 27). However, it is clear that the TCA in M2 macrophages is not affected, whereas FAO and OXPHOS are enhanced to generate ATP, and energy production remains constant.

Meanwhile, M2 macrophages metabolize lipids differently, undergoing OXPHOS and providing energy for cellular functions through the uptake of fatty acids. It has been suggested that the difference in lipid metabolism between the two phenotypes is due to their different functions. M2 macrophages are involved in the resolution, activated during the repair phase of functional activity of tissue injury, through OXPHOS and FAO as the main functional pathways (28). There is also evidence that FAO induces M2 phenotypic transformation, although whether this process is sufficient for M2 polarization has not been fully elucidated (29), inhibiting fatty acid transporter proteins promotes their M1 phenotype (30). Therefore, we are not yet able to determine the causal relationship between reprogramming of FAO energy utilization and M2 polarization behavior.

## Macrophage Metabolic Reprogramming-Mediated Regulation of Inflammatory Function

**Figure 1:**
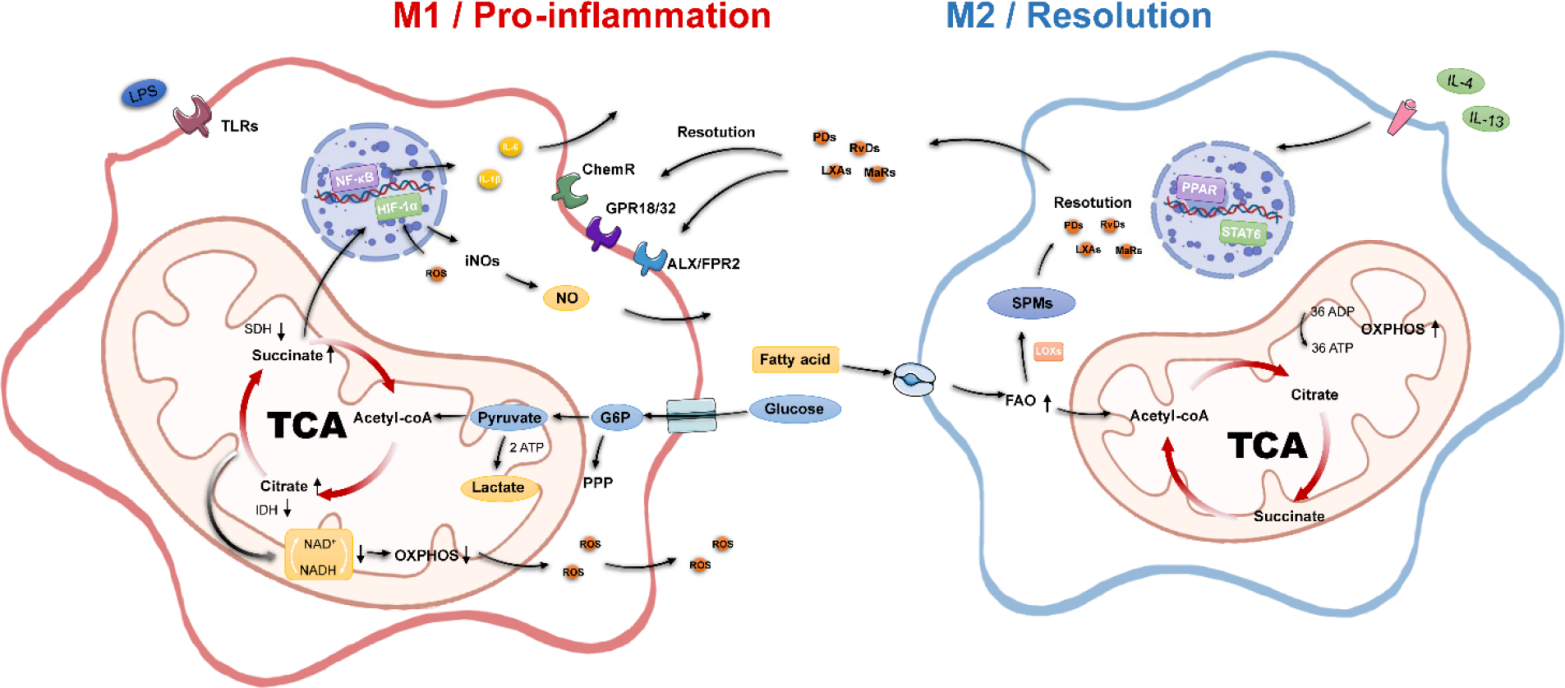
Metabolic and inflammatory characteristics of macrophages. M1 macrophages possess pro-inflammatory properties, disrupt the TCA, inhibit OXPHOS, undergo aerobic glycolytic processes, and activate multiple pro-inflammatory genes. M2 macrophages have pro-inflammatory properties; possess an intact tricarboxylic acid cycle, enhanced oxidative phosphorylation, and fatty acid oxidation processes; and secrete SPMs to promote inflammation resolution. Acetyl-CoA, acetyl-coenzyme A; ADP, adenosine diphosphate; ATP, adenosine triphosphate; FAO, fatty acid oxidation; G6P, glucose 6-phosphate; IDH, isocitrate dehydrogenase; NADH, nicotinamide adenine dinucleotide; OXPHOS, oxidative phosphorylation; PPP, pentose phosphate pathway; SDH, succinate dehydrogenase; SPMs, specialized pro-resolving mediators; TCA, tricarboxylic acid.

Because metabolic reprogramming is adapted to the inflammatory immune function of macrophages, intermediate and end products generated by energy metabolic reactions are involved in the inflammatory response of cells. The regulatory pathways are multifaceted, including membrane receptor–ligand activation during the initiation phase of metabolic reprogramming, activation of inflammation-associated nuclear transcription factors, secretion of inflammatory factor proteins, and production of inflammatory mediators (**Figure 1**).

The high sensitivity of macrophages to the tissue environment derives from their diverse signaling sensing system, where different receptors can mediate different downstream effects, starting from macrophages sensing inflammatory signals, the most classical is the M1 polarization response pathway of toll-like receptor TLR4 and its ligand LPS or IFN-γ. We mentioned earlier that M1 macrophage polarization involves inflammation-related metabolic reprogramming effects. The key feature of inflammatory macrophages is the acceleration of glucose transport uptake for aerobic glycolysis through upregulation of the glucose uptake transporter protein glucose transporter 1 (GULT1) to meet the process of rapid ATP production.

The accumulation of pyruvate-generated lactate, the inhibition of the TCA cycle, and the massive accumulation of citric and succinic acids directly mediated the upregulation of intracellular NO, ROS, and PGE2 levels (31). The upregulation of ROS is due to the inhibition of mitochondrial complex I by succinate overload (2), whereas the production of ROS stabilizes the hypoxia-inducible factor 1α (HIF-1α), which can further translocate to the nucleus to bind to hypoxia-responsive genes to enhance the glycolytic response, again with positive feedback on GULT1-mediated glucose uptake (32). The increase in lactate due to aerobic glycolysis is essentially HIF-1α, elevating the expression of lactate dehydrogenase. HIF-1α could be a linking target for energy metabolism and inflammatory response. HIF-1α is influenced by the integrity of the TCA, and its own stability promotes inflammatory responses. In LPS/IFN-γ–activated macrophages, HIF-1α can directly trigger IL-1β expression (33). Recently, it has also been proposed that stabilization of HIF-1α occurs late in the polarization of inflammatory macrophages and that early glycolytic production of lactate is not regulated by HIF-1α (34).

In addition, intracellular reactive ROS can also enhance the DNA binding capacity of p65 NF-κB to activate this pathway and enhance the transcription of pro-inflammatory genes. The expression of related inflammatory factors IL-1β, IL-6, and IL-12 further accelerates the M1 polarization process (35). As for the metabolism of substitution-activated M2 macrophages, the remarkable feature is the enhancement of the FAO process. Generally, exogenous IL-4 can induce M2 polarization. It is well known that the activation of the PPAR-γ/PGC-1β pathway promotes FAO and mitochondrial function, and the nuclear transcription factor STAT6 plays a certain role in it (36, 37). PPAR-γ, PGC-1β, and STAT6 can act as marker proteins and effectors of metabolic changes in M2 type. M2 macrophages are usually associated with enhanced phagocytosis, tissue repair, and anti-inflammatory effects. The polarization process from M1 to M2 is mostly also influenced by the microenvironment inside and outside the cell, such as the production of lactate, the release of adenosine nucleosides, and the concentration of α-ketoglutarate in the TCA cycle (38).

In addition, from the level of the immune regulation process, macrophage metabolism is endowed with phagocytosis of foreign substances, killing antigens and presenting antigens to T cells for subsequent immune responses of the body. At this time, macrophages will change the expression and secretion of inflammatory factors and related cellular mediators at multiple levels. As markers of M1 macrophage polarization, they will express cell membrane marker proteins CD80, CD86, and the major histocompatibility complex class II, corresponding to its bactericidal, antitumor, and Th1 cell responses (39, 40); in contrast, M2 macrophages express a variety of surface markers, including CD206, CD36, IL1Ra, and CD163, and release cytokines, such as IL-10, VEGF, and TGF-β (41).

Recent studies have proposed the concept of macrophage specialized pro-resolving mediators (42, 43). Such inflammatory mediators take arachidonic acid, docosahexaenoic acid, and eicosapentaenoic acids as substrates through exogenous uptake of ω-6, ω-3 unsaturated fatty acids, and fatty acid synthesis. Then, it is synergistically regulated by cyclooxygenases, lipoxygenases, and cytochrome P450 metabolic enzymes. Four classes of these mediators have been identified—resolvins, lipoxins, protectins, and maresins—with their representative resolution effects. Autocrine and paracrine secretion of this class of cellular mediators also positively affects the tissue environment in which macrophages reside. They can bind ALX/FPR2, GPR18, chemerine, and other macrophage surface membrane receptors (44), enhance macrophage cytokinesis, promote M2 phenotype transformation, and reduce the release of pro-inflammatory factors (45).

## Therapeutic Potential of Targeted Macrophage Metabolic Reprogramming in The Regulation of Inflammation in Ischemic Heart Disease

The role of macrophages in ischemic heart disease has been noted in recent years (46). Although immune cells account for only 10% of the entire cardiac tissue (47), circulating recruited and tissue-resident macrophages are able to modulate inflammatory effects through cell polarization and metabolic reprogramming, which then further influence tissue damage in the acute phase of heart failure as well as functional remodeling in the repair phase.

To discuss the effect of macrophage metabolic reprogramming on inflammation in ischemic heart disease, it is first necessary to clarify the temporal impact of macrophages in the occurrence and development of heart failure. Essentially, it is an inflammatory balancing effect exhibited by the functional focus of M1 and M2 cells in the acute and repair phases. Inflammatory activation in acute ischemic heart disease is caused by the DAMPs, and then, macrophages undergo metabolic reprogramming to the M1 phenotype to adapt to the pro-inflammatory profile at this time (48, 49). This type is usually induced by Ly6C^high^ monocytes recruited from peripheral circulation (50). In addition, it also secretes chemokines to recruit more macrophages from the spleen and circulating blood to participate in the regulation of inflammation in the heart (48), and this process occurs approximately 24 h to 7 days after myocardial ischemia (51). The key to the metabolic reprogramming of macrophages lies in a complex crosstalk environment in the context of ischemic injury to cardiac tissue (52). Different from the inflammatory activation conditions of macrophages in *in vitro* experiments, due to the local ischemia of the injured tissue, macrophages are in a hypoxic internal environment, and the HIF-1α is activated. It directly enhances glycolysis in macrophages, interrupting the TCA and the OXPHOS process, accompanied by increased citric acid and succinic acid production and lactic acid accumulation, thus stimulating a burst of downstream inflammatory factors, such as ROS (33, 53), activation of NF-κB pathway, and upregulation of IL-1β, IL-6, IL-18, and TNF-α (54–58). In turn, the secretion of various inflammatory factors will further promote M1-type polarization, leading to HIF-1α stabilization, resulting in the vicious cycle. Although this innate anti-inflammatory effect is necessary for the defense against bacterial infection, it may still cause excessive inflammatory injury to the myocardial tissue, whereas the inflammatory stimulus promotes excessive fibroblast activation, causing excessive myocardial fibrosis and affecting the left ventricular systolic-diastolic function (48, 56). This introduces the reparative effect of M2 macrophages during the repair phase, which, unlike M1 macrophages, promote inflammatory regression, enhance fragment phagocytosis, and promote the powerful effect of revascularization in myocardial ischemic injury (59). Activation of M2 macrophages in the heart overlaps in time with M1, typically at 4 to 14 days, produced by Ly6C^low^ monocytes (60). However, it has also been suggested that M2 is directly transformed by M1 macrophages generated by Ly6C^high^, and this part of the process is still controversial. Nonetheless, it is indisputable that M2 can promote local myocardial revascularization through the secretion of IL-10 to suppress inflammation and the release of the VEGF, thereby accelerating tissue repair (48, 61); this process continues until 14 days after the onset of ischemia.

## Concluding Remarks

Thus, the M1/M2 inflammatory behavior of macrophages and their metabolic reprogramming actions are crosstalk, enhanced cellular glycolysis, inhibition of the TCA, and weakened OXPHOS processes, leading to the production of inflammatory factor precursors, stabilization of HIF-1α structures, further production of potent bactericidal substances such as NO and ROS, and, ultimately, positive feedback on the activation of M1 macrophages. In contrast, the glycolysis-independent cell phenotype, through enhanced FAO and OXPHOS, was able to promote local wound healing and inflammatory regression of the tissue. Overall, on the basis of the conditions required for metabolic processes, enhancing FAO and OXPHOS in the local group of the heart, restoring local energy supply, and reducing progressive inflammatory effects triggered by M1-aerobic glycolytic may be the key points of stabilizing the inflammatory balance, as well as the metabolic balance, in ischemic heart disease.

## Author Contributions

XS wrote the article and made the figure. YL, QD, YH, and JD proofread the manuscript. WW, YW, and CL reviewed the article. All authors contributed to the article and approved the submitted version.

## Funding

This work was supported by the National Natural Science Foundation of China (Nos. 82174215 and 82174364), Major New Drug Creation of Ministry of Science and Technology (No. 2019ZX09201004–001-011), the Fundamental Research Funds for the Central Universities (Distinguished project), and Excellent Young Scientist Foundation of BUCM (BUCM-2019-JCRC005).

## Conflict of Interest

The authors declare that the research was conducted in the absence of any commercial or financial relationships that could be construed as a potential conflict of interest.

## Publisher’s Note

All claims expressed in this article are solely those of the authors and do not necessarily represent those of their affiliated organizations, or those of the publisher, the editors and the reviewers. Any product that may be evaluated in this article, or claim that may be made by its manufacturer, is not guaranteed or endorsed by the publisher.
